# Successful management of bilateral orbital metastases from invasive lobular breast cancer with abemaciclib and letrozole: a case report and literature review

**DOI:** 10.3389/fonc.2024.1286910

**Published:** 2024-01-23

**Authors:** Nuno Rodrigues Alves, Ana Filipa Duarte, David Fernandes Ribeiro, Rita Sousa Silva, Bruno Almeida Carvalho, Diogo Alpuim Costa

**Affiliations:** ^1^ Department of Ophthalmology, Centro Hospitalar Universitário Lisboa Central, Lisbon, Portugal; ^2^ Department of Ophthalmology, Unidade Local de Saúde de São José, Lisbon, Portugal; ^3^ Department of Ophthalmology, Hospital CUF Descobertas, Lisbon, Portugal; ^4^ Department of Anatomical Pathology, Hospital de Montilla, Andaluzia, Spain; ^5^ Department of Ophthalmology, Hospital Lusíadas de Lisboa, Lisbon, Portugal; ^6^ Deparment of Ophthalmology, Clínica de São João de Deus, Lisbon, Portugal; ^7^ Department of Haematology and Oncology, CUF Oncologia, Lisbon, Portugal; ^8^ Department of Medical Oncology, Hospital de Cascais, Cascais, Portugal; ^9^ NOVA Medical School (NMS), Faculdade de Ciências Médicas (FCM), Universidade NOVA de Lisboa (UNL), Lisbon, Portugal; ^10^ Department of Medical Oncology, AIM Cancer Center, Lisbon, Portugal

**Keywords:** CDK4/6 inhibitor, abemaciclib, letrozole, breast cancer, orbit, metastases, case report, review

## Abstract

Breast cancer is a significant global health concern, contributing to substantial morbidity and mortality among women. Hormone receptor-positive (HR+)/HER2-negative (HER2-) breast cancer constitutes a considerable proportion of cases, and significant advancements have been made in its management. CDK4/6 inhibitors (CDK4/6is) are a new targeted therapy that has demonstrated efficacy in adjuvant, advanced and metastatic settings. The propensity of lobular breast carcinomas for estrogen-rich sites, such as periocular tissues and orbital fat, may explain their tendency for orbital metastases. Current treatment strategies for these cases are predominantly palliative, and the prognosis remains poor. This article presents a unique case of a 51-year-old female with progressive right periorbital edema, pain, and limited ocular motility. An imaging work-up showed bilateral intra and extraconal orbital infiltration, which was biopsied. The histopathologic analysis disclosed mild chronic inflammatory infiltrate with thickened fibrous tissue and moderately differentiated lobular carcinoma cells, positive for GATA3 and CK7 markers, with 100% of tumor nuclei expressing estrogen receptors (ER+). A systemic evaluation showed a multicentric nodular formation in both breasts. Further diagnostic assessments unveiled an HR+/HER2- bilateral lobular breast carcinoma with synchronous bilateral orbital metastases. Systemic treatment was initiated with abemaciclib 150mg twice daily and letrozole 2.5mg once a day. However, this regimen was interrupted due to toxicity. After two weeks, treatment was resumed with a reduced abemaciclib dose (100mg twice daily) alongside letrozole, with a reasonable tolerance. Nearly two years after the initial diagnosis of inoperable metastatic cancer, the patient remains on the same systemic treatment regimen with no signs of invasive disease. This case report is the first of a patient presenting with bilateral orbital metastases from bilateral lobular breast cancer, showing an impressive and sustained response to a first-line treatment regimen combining abemaciclib and letrozole. A literature review on bilateral orbital metastases from breast cancer is also presented.

## 1 Introduction

Breast cancer is the most commonly diagnosed cancer globally and is the primary cause of cancer-related mortality in women (1). Categorized by disease stage and histological features, which include morphology and receptor status, breast cancer heterogeneity plays a crucial role in clinical decision-making (2, 3). Hormone receptor-positive (HR+)/HER2-negative (HER2-) breast cancer constitutes the most prevalent subtype, accounting for around 65% of cases (4). Another shared characteristic in luminal HER2- breast cancer is the hyperactivity of the CDK4/6 pathway, which contributes to resistance against endocrine therapy (5).

In recent years, significant strides have been made in the management of HR+/HER2- breast cancer through the introduction of CDK4/6 inhibitors (CDK4/6is), such as palbociclib, ribociclib, and abemaciclib, thereby improving outcomes for adjuvant, advanced and/or metastatic settings (6–15). CDK4/6is can block retinoblastoma protein hyper-phosphorylation, inducing G1 arrest and curtailing proliferation (16, 17). A novel therapeutic approach by abemaciclib (Verzenio; Eli Lilly), an oral selective small molecule targeting the CDK-RB1-E2F pathway pivotal for cell cycle progression, has garnered substantial attention (16). The MONARCH 3 trial, a phase 3, double-blind, randomized study, recently demonstrated that abemaciclib plus nonsteroidal aromatase inhibitor (NSAI - including letrozole) resulted in more prolonged overall survival compared to placebo plus NSAI (absolute improvement of 13.1 months) (hazard ratio, 0.804; 95% CI, 0.637 to 1.015; p= 0.0664; p-value did not reach threshold for statistical significance) and significantly extended progression-free survival (hazard ratio, 0.535; 95% CI, 0.429 to 0.668; p= <0.0001; 29.0 months in the abemaciclib arm, 14.8 months in the placebo arm) (10, 18). Consequently, combining CDK4/6is with endocrine therapy emerged as one of the preferred regimens for patients with advanced and/or metastatic HR+/HER2- breast cancer.

Furthermore, abemaciclib distinguishes itself as the sole CDK4/6 inhibitor examined in a dedicated clinical trial specifically addressing metastatic disease within the central nervous system (CNS) (NCT02308020, phase II trial, encompassing leptomeningeal disease, a criterion indicative of greater severity) (19, 20). In contrast, trials involving palbociclib and ribociclib had limited inclusion or lacked representation of patients with disease at this CNS level (21–23).

To our knowledge, we report the first clinical case of bilateral orbital metastases as the presenting feature of bilateral breast cancer treated with a CDK4/6i and an aromatase inhibitor.

## 2 Case report

**Figure 1:**
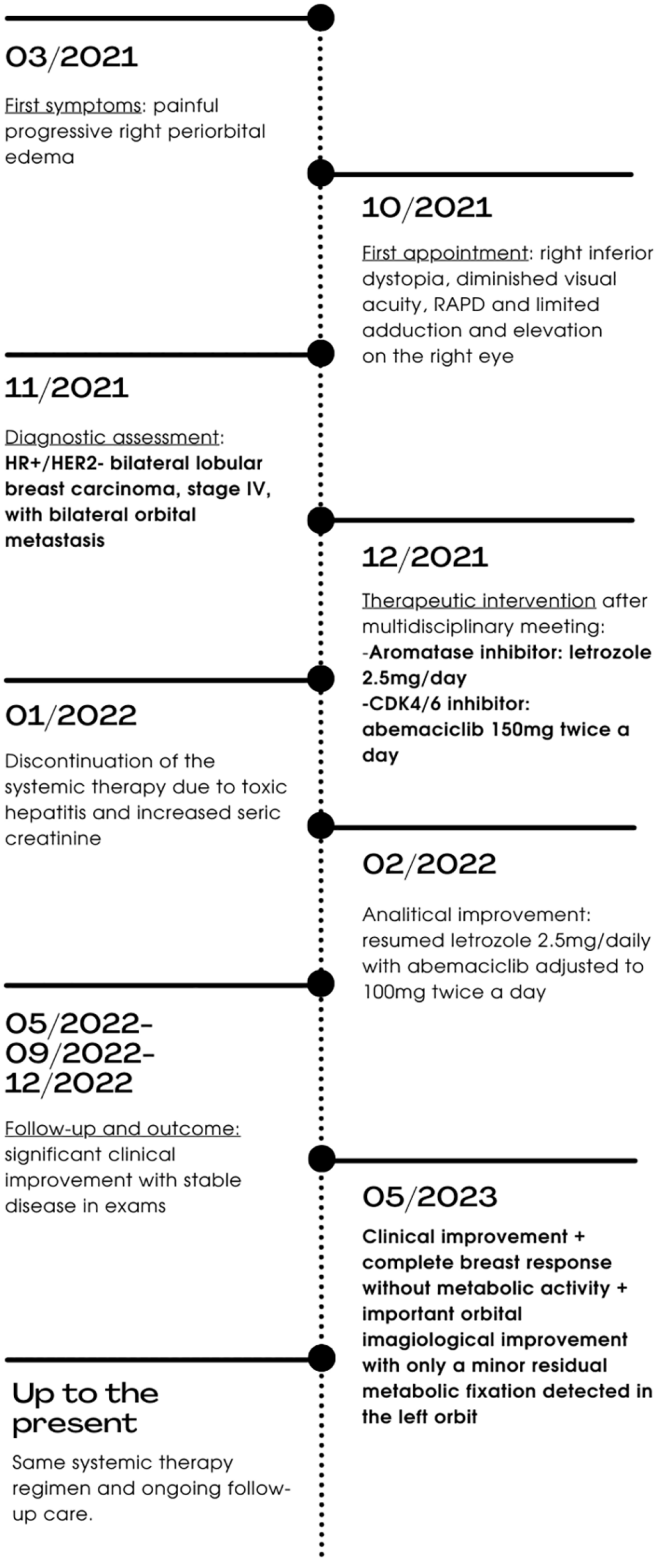
Patient timeline.

**Figure 2:**
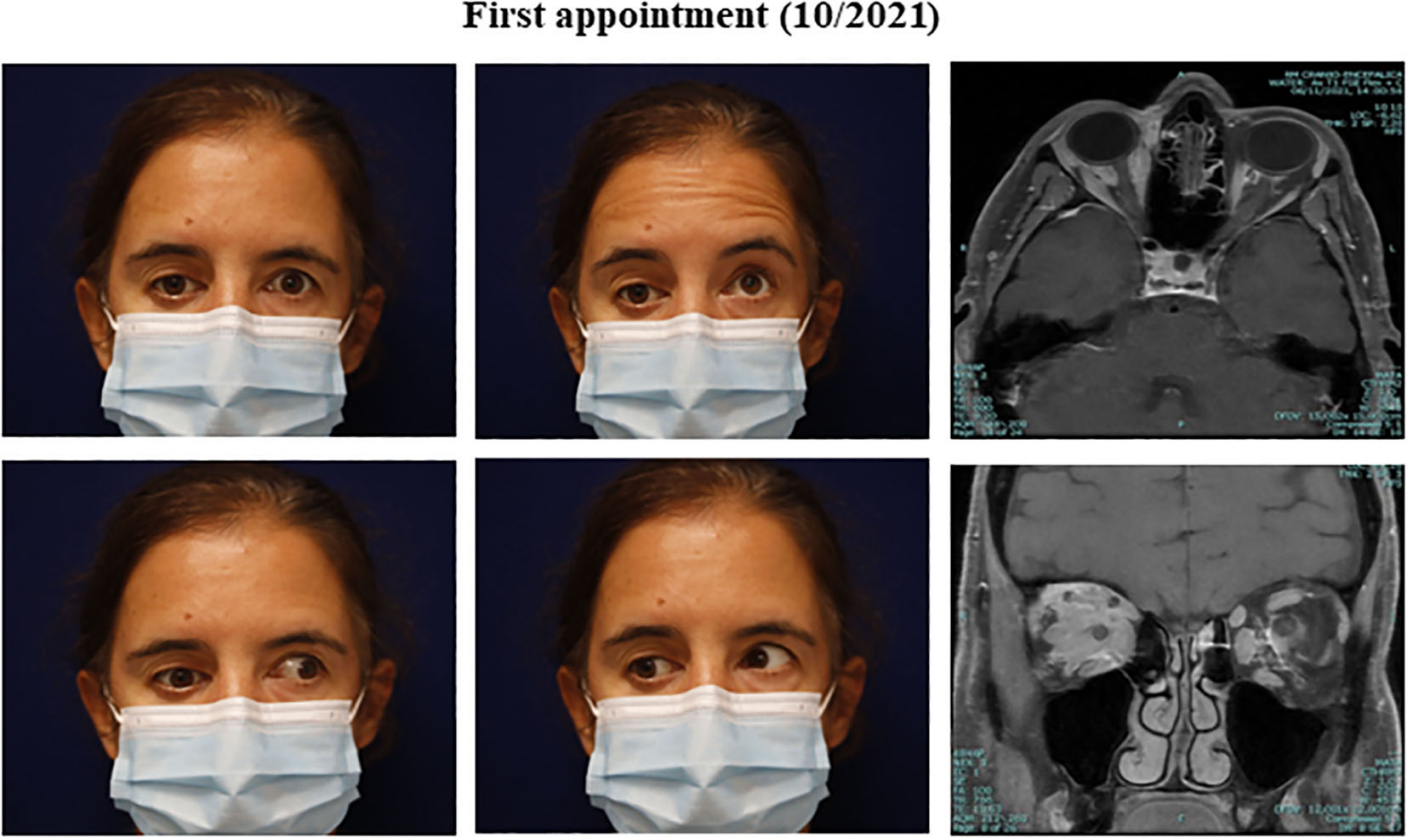
Clinical presentation and orbital findings at the initial appointment. (Clinical pictures) Right inferior dystopia with restriction in extraocular elevation and adduction of the right eye. (Orbit Imaging) Orbit axial and coronal T1 MRI showing post-gadolinium enhancing lesions (intra and extra-conal), with mass effect and inflammatory changes of orbital fat.

A 51-year-old female presented with a seven-month history of painful progressive periorbital edema and limitation of extraocular movements of the right eye (**Figure 1**). Her medical history revealed essential hypertension, dyslipidemia, adenomyosis, benign thyroid nodule, gallbladder polyp, major depression, allergic rhinitis, and a smoking history of 32 pack-years. Her pharmacological regimen included candesartan, rosuvastatin, montelukast, paroxetine, lorazepam, mirtazapine, and bupropion. Additionally, she reported an allergy to ibuprofen and had a pertinent family history of prostate cancer in two brothers, diagnosed at 64 and 70 years old. Physical examination revealed inferior dystopia of the right eye with limited horizontal movements on the right eye, without diplopia (**Figure 2**). The best corrected visual acuity was 20/30 right eye (OD) and 20/20 left eye (OS), Ishihara test 6/10 OD vs. 9/10 OS, and a relative afferent pupillary defect (RAPD) was detected on the right eye. Hertel exophthalmometry showed mild asymmetry of 15mm OD and 16mm OS. In the visual fields, there was an inferonasal paracentral scotoma in the right eye, while the left eye had a normal visual field. Optical coherence tomography (OCT) indicated a mild optic disc edema and a reduction in retinal ganglion cell layer thickness in the right eye, without changes in the nerve fiber layer; no changes were observed in the left eye. Biomicroscopy, intraocular pressure, and ocular fundoscopy findings were unremarkable.

### 2.1 Diagnostic assessment

After the initial presentation, an orbital and cranial magnetic resonance image (MRI) was requested. An extensive intra- and extraconal orbital infiltration involving the optic nerve, extrinsic ocular musculature, and lacrimal gland was found on the right orbit. Similar discrete signal alterations were identified within the left orbit, mainly between the optic nerve and the medial and inferior rectus muscles (**Figure 2**).

**Figure 3:**
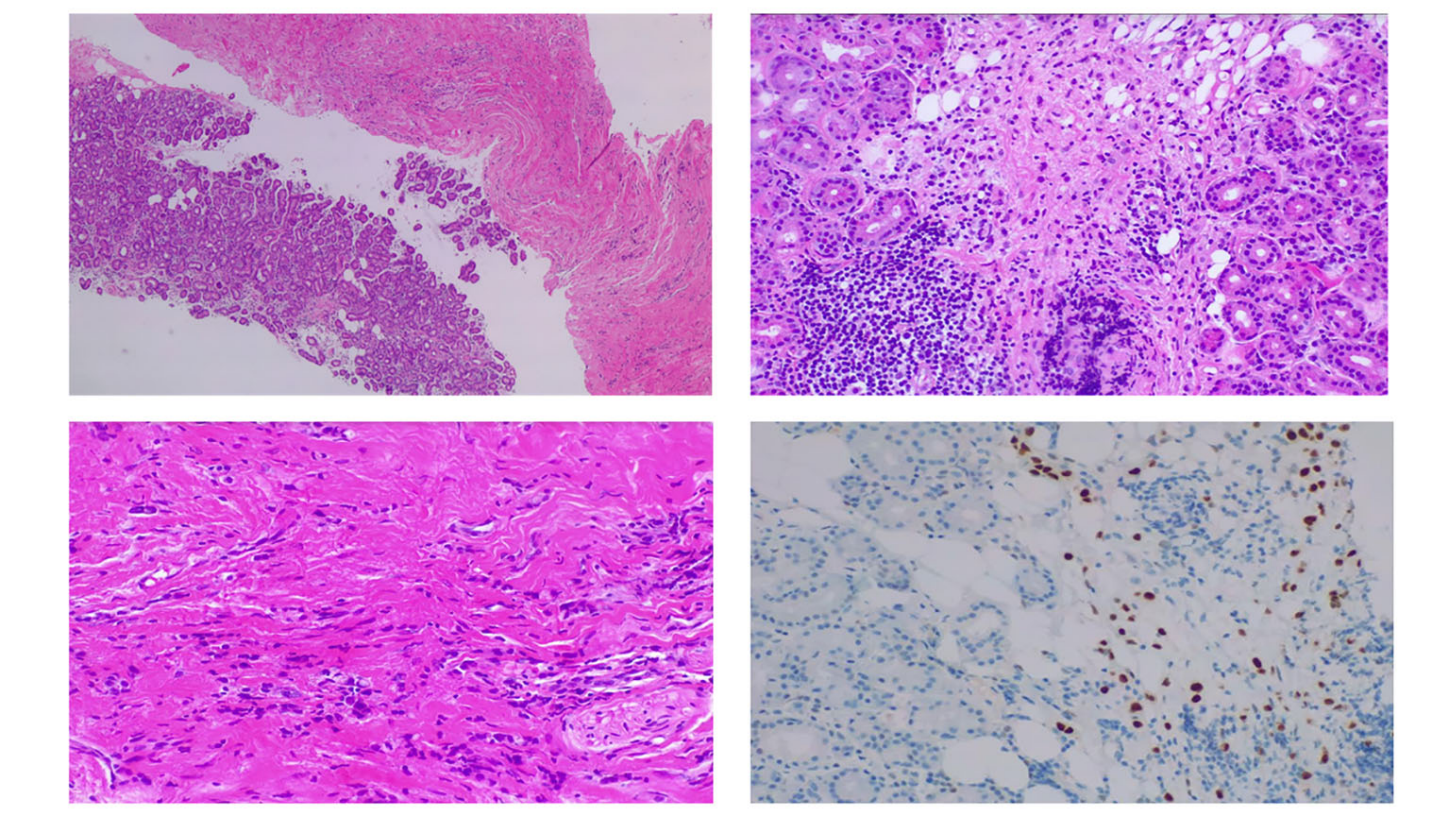
Orbital biopsy. (Supero-left) Orbital biopsy comprised soft tissue and lacrimal gland fragments with infiltration by lobular breast carcinoma. (Supero-right) Lacrimal gland showing discohesive cells with nuclear atypia, many resembling signet-ring cells and containing intracellular mucin. (Infero-left) Thickened fibrous tissue where isolated cells and cell rows of similar histologic characteristics are identified. (Infero-right) Infiltrating cells exhibiting immunoreactivity for estrogen receptors, suggesting breast origin.

An incisional biopsy of the right orbit was performed, which included several samples collected from the superior and superior-temporal areas through a lid cease approach. Histopathologic examination revealed moderately differentiated lobular carcinoma cells (**Figure 3**). Immunohistochemical analysis revealed positivity for GATA3 and CK7 markers, with 100% of tumor nuclei expressing estrogen receptors (ER+) (**Figure 3**). The c-ERB-B2 (HER2/neu) score was 0, and E-cadherin and PD-L1 (combined positive score) expressions were negative.

Following these findings, a comprehensive work-up was initiated to identify the primary tumor. This encompassed breast ultrasonography, mammography, breast MRI, esophagogastroduodenoscopy, gynecological transvaginal ultrasound, lumbar puncture, and positron emission tomography (PET)/CT scan employing 18-fluorodeoxyglucose (18F-FDG). The PET/CT 18-FDG scan revealed moderate heterogeneous radiopharmaceutical uptake in both orbits, the right axillary lymph node, and mild to moderate metabolic activity in the stomach and uterus. Esophagogastroduodenoscopy uncovered hyperemic gastropathy without neoplastic or dysplastic tissue, and transvaginal ultrasonography identified adenomyosis and leiomyomas. A lumbar puncture revealed suspected neoplastic cells, prompting a neuroaxis MRI that showed no suspected invasive disease. Breast imaging unveiled multicentric nodular formations. These solid, irregularly contoured nodules numbered at least three on the right and two on the left, with a larger, coarser superior-external nodule on the right (10mm) along with notably enhancing right axillary lymph nodes, the largest measuring 19 mm. Given the suspicious nature of the findings in both breasts (BI-RADS Category 4), ultrasound-guided core biopsies were performed on two breast nodules and the right axillary node. Histological analysis revealed invasive carcinoma with a lobular pattern, moderately differentiated (Grade 2). ER was positive in 90% of cells, while progesterone receptor (PR) was 100%. HER2 was negative, as was E-cadherin. The dominant lesion in the right breast exhibited a proliferation index (Ki67) of 10%, and in the left breast, it was 7%. Axillary cytology confirmed these findings.

Hence, the patient was diagnosed with metastatic lobular breast cancer, classified as stage IV disease according to the AJCC 8th edition TNM staging (24). The case was discussed in a multidisciplinary breast cancer tumor board, and, given the metastatic and unresectable nature of the disease, coupled with its unsuitability for local intervention, it was decided to initiate systemic treatment with a CDK4/6i plus an aromatase inhibitor.

### 2.2 Therapeutic intervention

In December of 2021, based on the results of the MONARCH 3 clinical trial (10), the patient initiated abemaciclib 150mg twice daily, combined with letrozole 2.5mg once a day.

### 2.3 Follow-up and outcome

In January 2022, just a month after starting systemic therapy, the patient developed analytical toxic hepatitis, marked by elevated ALT and AST levels at grade 3, along with GGT elevation at grade 4, as classified by the Common Terminology Criteria for Adverse Events (CTCAE) (25), which contributed to the temporary withdrawal of treatment. Furthermore, this was accompanied by increased serum creatinine (grade 2). After a two-week interval, during which laboratory parameters were reassessed and showed progressive improvement, the patient resumed letrozole, while the dose of abemaciclib was adjusted to 100mg twice a day. Close monitoring of laboratory values was undertaken. Over the subsequent four months, there was a gradual recovery in hepatic parameters, although the serum creatinine level remained at grade 1.

Concomitantly, the patient encountered grade 1 diarrhea, nausea, and asthenia. While adverse effects progressively resolved, grade 1 diarrhea persisted and was effectively managed through interventions such as loperamide administration, oral hydration, and dietary adjustments.

During follow-up, the patient exhibited a marked clinical response to treatment, with significant recovery of visual acuity and extraocular motility, which occurred as early as the first cycle of abemaciclib and continued despite the reduced dosage of 100mg twice daily. The patient underwent repeated orbital MRI, breast MRI, and PET/CT with 18F-FDG imaging, confirming a favorable response, with bilateral tumor size reduction on both orbits and breast areas, without new lesions.

**Figure 4:**
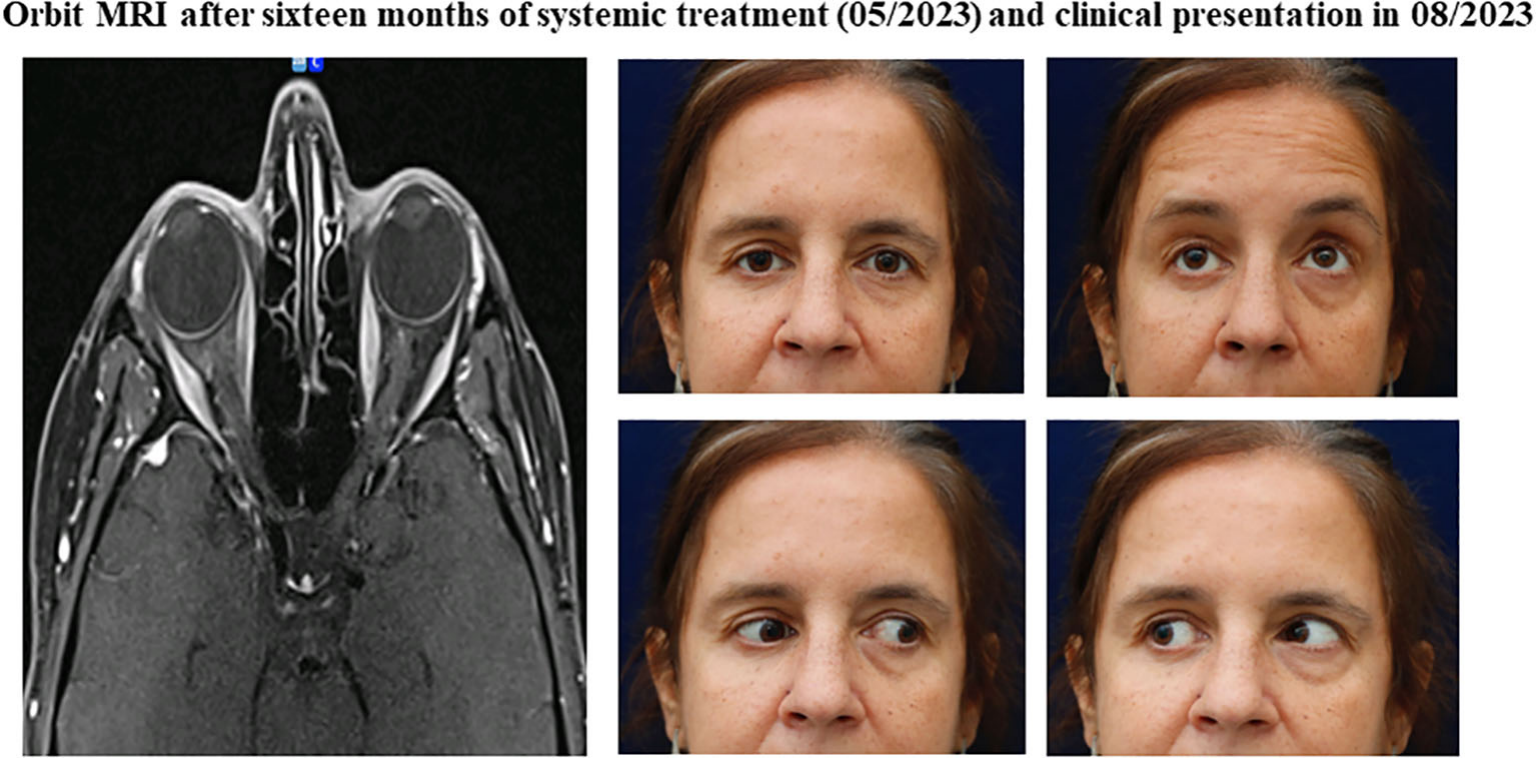
Orbital findings after sixteen months of systemic treatment and clinical presentation in 08/2023. (Orbit Imaging) Orbit axial and coronal T1 MRI showing imaging improvement in the orbital region, marked by permeabilization of bilateral intraorbital fat. (Clinical pictures) Significant clinical improvement in ocular movement restrictions, with only partial limitation remaining on right adduction.

In May 2023, after sixteen months of systemic therapy, the patient achieved a complete response in both breasts and a significant improvement on orbital imaging, with practically complete permeabilization of bilateral intraorbital fat with only a minor residual metabolic fixation detected in the left orbit (**Figure 4**). Visual acuity remained stable at 20/20 OI, with visual field recovery, and extraocular motility improved with only a mild limitation of right eye adduction (**Figure 4**). OCT revealed an improvement in retinal ganglion cell layer thickness and normalization of the optic disc in the right eye. Hertel exophthalmometry was 14mm OD and 15mm OS, while the rest of the physical examination yielded unremarkable findings.

The patient continues to adhere to the same systemic therapy regimen, remains resilient with her progress, and actively participates in follow-up care. In the last follow-up, the patient resumed her professional and social activities, not reporting any limitations in daily tasks.

## 3 Discussion and conclusion

Orbital metastases represent a complex subset, accounting for 1–13% of all orbital neoplasms and affecting around 2–5% of patients diagnosed with systemic malignancies (26). Notably, breast cancer (36%), melanoma (10%), and prostate cancer (8.5%) emerge as the most common primary sources of orbital metastases (27–29). They are typically unilateral, but clinically evident bilateral metastases are reported in 4–20% of cases (30). They are often identified after the primary tumor diagnosis, with a prevailing interval of 3 to 6 years (31, 32). However, exceptional cases have revealed latency extending over decades post-cancer diagnosis, the longest being 42 years after the primary breast carcinoma identification (33). The median age of orbital metastases from breast cancer is 54 (range 28-77 years) (26, 29).

Various tumors and tumor-like lesions can involve the orbit (34), making imaging a crucial step in the initial differential diagnosis of patients with new symptoms or without a previous diagnosis (35). Thyroid eye disease, granulomatosis with polyangiitis, amyloidosis, sarcoidosis, lymphoproliferative disease, orbital inflammatory pseudotumor, IgG4-related disease, as well as solid tumors, infectious and vascular conditions, are always important to consider when radiologic changes are found in the orbital space (36). A biopsy is warranted when clinicoradiologic findings are inconclusive or a previous histological diagnosis is questioned (36, 37).

Intriguingly, orbital metastases can occasionally serve as the inaugural finding of an undetected primary tumor, appearing in an estimated 10% to 31% of cases (31, 38, 39). Considering histological subtypes, lobular breast carcinoma, comprising 10-15% of all breast cancer cases (40), exhibits an increased expression of ER and PR but has decreased HER2 positivity compared to the no special type (NST)/ductal carcinoma (41). In contrast, E-cadherin expression in ductal breast carcinoma limits cellular dispersion, and therefore, orbital metastases from NST are rare (42). Conversely, it is worth noting the propensity of lobular carcinomas for metastases to sites with a substantial supply of estrogen, such as the gastrointestinal and genitourinary tracts (42–47). This could be attributed to the steroid hormone production in periocular tissues and orbital fat, fostering a conducive milieu to metastases of lobular breast carcinoma (45–47).

Despite advances, therapeutic strategies for managing orbital metastases remain a challenge due to the scarcity of data. Current treatment approaches generally lean toward a palliative plan, especially as orbital metastases from breast cancer often arise in the context of advanced end-stage disease (48). Even with treatment, the prognosis for patients diagnosed with orbital metastases yields a mean survival of 31 months (1-116 months) (31, 49).

A review of cases involving bilateral orbital metastases from breast cancer, as reported in English-language literature, was conducted through PubMed, Medline, and Google Scholar databases using the appropriate controlled [MESH] keywords “breast cancer”, “bilateral”, “metastases”, “ocular” and “orbit” and acknowledging references list. The selected articles included case reports and case series that provided detailed clinical, histological, and treatment descriptions (30, 42, 47, 48, 50–77). The summarized findings are presented in **Table 1**. Forty-two patients, mostly females (95%), were found. The mean age was 59 years (ranging from 36 to 83 years). The majority (64%) had known breast cancer (42, 47, 50, 53, 57, 58, 61, 62, 64–68, 70–73, 76, 77), and orbital metastases were usually identified around 4.8 years after the first diagnosis. Due to the anatomical constraints of the compact orbit space, these metastases usually present as space-occupying lesions, leading to significant clinical symptoms (31). Affected patients commonly exhibit limited ocular motility (55%) (30, 48, 50, 51, 54–58, 60, 61, 64, 65, 67–71, 73–77), vision loss (29%) (51, 53, 54, 56, 57, 59, 63, 64, 68, 71, 75, 77), periorbital edema (24%) (30, 52, 53, 59, 62, 65, 66, 68, 69, 76), diplopia (21%) (48, 55, 57, 58, 64, 67, 70, 71, 73), proptosis (14%) (47, 51–53, 56, 59), ptosis (14%) (53, 54, 69, 73, 75, 77), palpable mass (7%) (54, 63, 70), as well as dystopia (64, 74), and upper lid retraction (53, 67) (both 5%). A notable and intriguing occurrence is enophthalmos, observed in 2 cases (5%) (61, 75). This is likely due to the infiltration of neoplastic cells into the extraocular muscles and retro-bulbar stromal tissues, leading to desmoplasia, fibrosis, and the retraction of the eye globe (78). The majority of orbital metastases exhibit lobular histology (50%) (30, 42, 47, 52, 53, 55, 57, 58, 60, 62, 65–67, 69, 72, 76, 77) vs. ductal (14%) (56, 59, 64, 71, 72, 75) (48, 61, 74) vs. mixed (5%) (72), a trend that is consistent throughout existing literature (42). The immunophenotype of these clinical cases is predominantly hormone receptor-positive in breast cancer, specifically belonging to the luminal subtype (42, 47, 56, 62, 64–66, 69, 72, 76). These metastases often demonstrate a diffuse infiltration pattern within the orbit, affecting bones and extraocular muscles. Invasion of intracranial structures is rare, with brain metastases identified in only 6 cases (14%) (50, 63, 68, 72). Despite various forms of palliative treatment, bilateral orbital metastasis from breast cancer remains a poor prognostic factor, with a mean survival of 16 months following diagnosis (range 0.5 to 41 months) (31).

**Table 1:** Literature review of 42 clinical cases of bilateral orbital metastases from breast cancer.

Source	Known previous disease (Y/N)	Ophthalmologic presentation	Other metastatic sites	Orbital imaging at presentation	Histology	Immunophenotype	Treatment	Outcome
**Bedford 1960** (50)	Y	Limited ocular motility	Liver, peritoneal carcinomatosis, lymph nodes, skin, brain	NA	NA	NA	NA	Deceased after 2 weeks
**Capone 1990** (51)	N	Proptosis, limited ocular motility, pain, vision loss	None	Bilateral diffuse EOM enlargement	NA	NA	Refused treatment	Deceased after 23 months
**Glazer 1991** (52)	N	Proptosis, periorbital edema	None	Bilateral enlargement of multiple EOM and anterior soft tissue infiltration	Lobular	NA	RT, HT (tamoxifen) and CHT	Progression (multiple metastases after 1 year)
**Rhatigan 1995** (53)	Y	Proptosis, ptosis, periorbital edema, vision loss, upper lid retraction	NA	Soft tissue masses encasing the globes	Lobular	NA	RT	Deceased after 2 weeks
**Po 1996** (54)	N	Limited ocular motility, ptosis, palpable mass, vision loss	None	Bilateral diffuse infiltration	NA	NA	CHT	Deceased after 5 months
**Zambarakjj 1997** (55)	N	Limited ocular motility, diplopia	Cerebrospinal fluid	Ill-defined ‘cuffing’ of the globe, optic nerve and EOM	Lobular	NA	RT, CHT (cytarabine, intratectal methotrexate)	Improvement of symptoms
**Garcia 1998** (56)	N	Proptosis, limited ocular motility, vision loss	None	Bilateral diffuse infiltration	Ductal	Luminal B	RT, HT (tamoxifen)	Improvement of symptoms
**Toller 1998** (57)	Y	Limited ocular motility, pain, vision loss, diplopia	Bone, lymph nodes	Bilateral diffuse enlargement of EOM, infiltration of fat, Tenon capsule, sclera, and eyelid soft tissue	Lobular	NA	Refused treatment	Deceased after 9 months
**Lacey 1999** (58)	Y	Enophthalmos, limited ocular motility, diplopia	None	Bilateral nodular enlargement of MR and IR	Lobular	NA	NA	NA
**Stuntz 2000** (59)	N	Proptosis, pain, periorbital edema, pain, vision loss	Bone, visceral	Bilateral posterior mass lesions	Ductal	NA	RT, CHT, HT	Progression, deceased after 34 months
**Lell 2004** (30)	N	Periorbital edema, limited ocular motility	None	Bilateral diffuse infiltration of the EOM and extra and intraconal compartments	Lobular	NA	NA	NA
**Gonçalves 2005** (60)	N	Enophtalmos, limited ocular motility	None	Infiltration of both orbits	Lobular	NA	CHT (cyclophosphamide, adriamycin)	Improvement of symptoms and stable after 2 years of follow-up
**Spitzer 2005** (48)	N	Limited ocular motility, diplopia	None	Bilateral diffuse EOM enlargement	NA	NA	CHT (cyclophosphamide, doxorubicin) + HT (letrozole) + RT	Improvement of symptoms
**Peckham 2005** (61)	Y	Enophtalmos, limited ocular motility	NA	Bilateral thickening of all EOM sparing the anterior tendon.	NA	NA	NA	NA
**Kuchel 2006** (62)	Y	Periorbital edema	None	Bilateral inferior extraconal and intraconal mass lesions	Lobular	Luminal	NA	NA
**Gasperini 2007** (63)	N	Palpable mass, vision loss	Brain	Infiltration of orbital bone, both optic nerves and left orbital mass lesion lateral to the LR	NA	NA	RT, CHT and optic nerve sheath fenestration	improvement of symptoms
**Milman 2008** (64)	Y	Dystopia, limited ocular motility, vision loss, diplopia	Bone, lymph nodes, pancreas	Bilateral nodular enlargement of EOM	Ductal	Luminal	HT (letrozole, anastrozole)	Improvement of symptoms and diminished systemic metastases with letrozole, but progression after 10 months and switch to anastrozole. 15 months free from disease with anastrozole
**Kouvaris 2008** (65)	Y	Periorbital edema, limited ocular motility	Bone, lymph nodes, skin	Bilateral nodular enlargement of EOM	Lobular	Luminal A	RT, HT (anastrozole), CHT (vinorelbine, mitomycin), hyperthermia	Improvement of symptoms. Deceased after 13 months
**Jaspers 2009** (66)	Y	Periorbital edema	Bone, liver, peritoneal carcinomatosis	Bilateral mass lesions	Lobular	Luminal	CHT (docetaxel), RT	NA
**Murthy 2011** (67)	Y	Limited ocular motility, upper lid retraction, diplopia	Lungs	Bilateral diffuse EOM enlargement	Lobular	Triple negative	RT, HT (tamoxifen)	Improvement of symptoms and complete remission after 18 months of follow-up
**Kim 2011** (68)	Y	Limited ocular motility, pain, periorbital edema, vision loss	Bone, brain	Bilateral extraconal masses with periostieal thickening	NA	NA	RT	Improvement of symptoms. Deceased after 19 months
**Kim 2012** (69)	N	Periorbital edema, limited ocular motility, ptosis	Bone, lymph nodes	Bilateral soft tissue mass lesions molding to the globes	Lobular	Luminal	CHT + HT	Undergoing treatment at time of publication
**Wiggins 2012** (70)	Y	Palpable mass, limited ocular motility, diplopia	NA	Bilateral fusiform enlargement of the MR, LR muscles sparing the tendons	NA	NA	RT+HT	Improvement of symptoms. Deceased after 8 months
**Khan 2015** (71)	Y	Limited ocular motility, vision loss, diplopia	Bone	Mass lesions in the left IR and right LR	Ductal	NA	RT	Partial orbit response. Undergoing treatment at time of publication
**Raap 2015** (47)	Y	Proptosis	Liver	Ill-defined mass lesions	Lobular	Luminal	Right enucleation, CHT (paclitaxel)	NA
**Raap 2015** (47)	N	NA	NA	NA	NA	Luminal	NA	NA
**Jakobiec 2017** (42)	N	NA	NA	NA	Lobular	Triple negative	NA	NA
**Jakobiec 2017** (42)	Y	NA	NA	NA	Lobular	Luminal	NA	NA
**Blohmer 2020** (72)	Y	NA	Bone, brain, spleen	NA	Ductal	NA	Systemic treatment	Deceased after 2 months
**Blohmer 2020** (72)	N	NA	Bone	NA	Lobular	NA	RT, systemic treatment	Deceased after 41 months
**Blohmer 2020** (72)	Y	NA	Bone, lymph nodes	NA	Lobular	NA	RT, systemic treatment	Deceased after 12 months
**Blohmer 2020** (72)	Y	NA	None	NA	Lobular	NA	RT, systemic treatment	Deceased after 22 months
**Blohmer 2020** (72)	Y	NA	Bone	NA	Lobular	NA	RT, systemic treatment	Lost to follow-up
**Blohmer 2020** (72)	Y	NA	Rectum	NA	Mixed (lobular-ductal)	Luminal	RT, systemic treatment	Deceased after 25 months
**Blohmer 2020** (72)	Y	NA	Bone, brain	NA	Mixed (lobular-ductal)	NA	RT, systemic treatment	Deceased after 9 months
**Blohmer 2020** (72)	Y	NA	Peritoneal carcinomatosis, gastrointestinal	NA	NA	NA	RT, systemic treatment	Deceased after 4 months
**Blohmer 2020** (72)	Y	NA	Bone, brain	NA	NA	NA	RT, systemic treatment	Deceased after 10 months
**Marotta 2020** (73)	Y	Limited ocular motility, pain, ptosis, diplopia	Bone, pleural	Nodular enlargement of the left MR, IR and the right SR	NA	NA	CHT (trastuzumab, pertuzumab, docetaxel), RT	Improvement of symptoms
**Dimopoulos 2020** (74)	NA	Dystopia,limited ocular motility	NA	Bilateral EOM enlargement and unilateral intraconal mass	NA	NA	NA	NA
**Muhammad-Ikmal 2022** (75)	N	Enophthalmos, limited ocular motility, ptosis, vision loss	NA	Infiltrating mass invading the ethmoidal sinuses, frontal sinuses and both orbits	Ductal	NA	CHT+RT	Stable disease control after 1 year
**Tsutsui 2022** (76)	Y	Limited ocular motility, periorbital edema	Bone	Bilateral soft tissue, medial and retrobulbar infiltration	Lobular	Luminal A	HT (fulvestrant, abemaciclib)	Progression to orbital metastases.Deceased after 3 months
**Karimaghaei 2022** (77)	Y	Limited ocular motility, ptosis, vision loss	None	Bilateral retrobulbar infiltration	Lobular	NA	NA	NA

CHT (chemotherapy), EOM (extraocular muscle), F (female), HT (hormonotherapy), IR (inferior rectus), LR (lateral rectus), M (male), MR (medial rectus), N (no), NA (nonavailable), RT (radiotherapy), SR (superior rectus), and Y (yes).

The emergence of CDK4/6is, such as palbociclib, ribociclib, and abemaciclib, has brought a remarkable shift in the paradigm of the treatment of advanced and/or metastatic HR+/HER2- breast cancer (6–13). Notably, none of the included treatment guidelines name specific CDK4/6is treatments but recommend the class broadly, as there have been no head-to-head clinical trials to date comparing the three approved CDK4/6is, and the efficacy of each appears to be similar (22, 79, 80). Nevertheless, the latest comprehensive survival data imply possible distinctions between the different CDK4/6is, indicating a trend in preferred choices, as palbociclib did not increase overall survival (23).

Notably, abemaciclib has exhibited efficacy in managing intraocular metastases originating from breast cancer, as elucidated in two case reports (81, 82). A woman 57 years old with iris metastases, which regressed within four months and remained undetectable through an eight-month follow-up using a combination of abemaciclib and letrozole (82). In a second case, a woman in her 50s with bilateral choroid metastases stemming from breast cancer positively responded to abemaciclib and fulvestrant within four months after the beginning of treatment (81). The significant response observed to abemaciclib in treating intraocular metastases aligns with preclinical and clinical evidence showing its ability to penetrate the central nervous system (19, 20). This suggests that abemaciclib holds promise as a viable therapeutic option in this specific clinical scenario. No cases of orbital metastases treated with these targeted therapies were found.

To the best of our knowledge, we present the first case of a patient whose initial presentation had bilateral orbital metastases originating from bilateral lobular breast cancer with a substantial and dramatic response to a first-line treatment regimen that combined abemaciclib and letrozole.

Interestingly, our case report emphasizes that even with a reduced dose of 100mg, abemaciclib demonstrated efficacy without compromising the outcome. Similar to the MONALEESA trials, overall survival outcomes for patients with HR+/HER2- advanced breast cancer exhibited comparable results between those who underwent dose reductions of ribociclib and those who received the standard dose (83). This observation prompts the intriguing idea of tailoring treatment by personalizing doses for individual patients, considering their unique responses and tolerances.

Further comprehensive investigations are warranted to fully comprehend the potential of CDK4/6is in managing orbital metastases. It is essential to conduct rigorous studies that evaluate the safety and efficacy of different CDK4/6is through head-to-head comparisons and explore the impact of varying doses. These studies will provide valuable insights into optimizing treatment strategies and potentially improving outcomes for HR+/HER2- breast cancer patients with orbital metastases.

Therefore, the selection of CDK4/6i depends mainly on the toxicity profile and comorbidities of the patient. For instance, it is conceivable to avoid abemaciclib in patients with inflammatory bowel disease, while ribociclib should be avoided in patients with prolonged QT interval alterations on electrocardiogram (23). Conversely, palbociclib should be cautiously approached in patients with compromised bone marrow reserve (23).

Notably, the most frequent adverse effect observed during abemaciclib treatment is diarrhea, primarily of grade 1 severity (10), which aligns with our clinical case. Additionally, our patient exhibited analytical findings of hepatic toxicity and a mild increase in serum creatinine one month after initiating systemic treatment. These events, known and expected in the MONARCH trials (10), resolved upon withdrawal and subsequent reduction of abemaciclib dosage to 100mg twice a day. Adverse events and toxicities have been recognized in certain instances to correlate with positive treatment outcomes in cancer therapy (84, 85). Nevertheless, the current understanding of predictive factors for response to available breast cancer treatments remains insufficient. This uncertainty prompts the exploration of unconventional factors, such as the microbiota’s role in offering insights into individual risk and prognosis, pharmacokinetics, pharmacodynamics, and clinical efficacy (86, 87). Recent research has demonstrated the capacity of the gut microbiota to influence the effectiveness and adverse effects of cancer treatments, as both cancer and anticancer therapies have bidirectional interactions with gut microbiota (86, 88, 89).

While the correlation is intriguing, it is essential to acknowledge that it may not be straightforward. The connection between adverse effects and treatment response can be intricate, influenced by various patient-specific elements, tumor characteristics, and the complex interplay of the drug with the body’s physiological systems. Thus, while a correlation between diarrhea, changes in hepatic parameters, and treatment response in breast cancer with abemaciclib is captivating, further investigations are imperative to establish a causal relationship and unveil the underlying mechanisms linking these observations.

As we navigate these investigations, we must recognize the limitations inherent in single-case reports and exercise caution in extrapolating results to similar presentations and the long-term effects that may extend beyond sixteen months.

In conclusion, this clinical case underscores the potential of combining CDK4/6is, especially abemaciclib, with endocrine therapy in treating HR+/HER2- orbital metastatic breast cancer. While this case report highlights promising therapeutic avenues, it underscores the need for comprehensive studies, acknowledging the complexities of individual responses and the influence of factors like microbiota. As we advance toward more personalized oncology approaches, these findings encourage us to delve deeper into the interplay between treatments, adverse effects, and patient outcomes to optimize therapeutic strategies in metastatic breast cancer.

## Data availability statement

The original contributions presented in the study are included in the article/supplementary material. Further inquiries can be directed to the corresponding authors.

## Ethics statement

Written informed consent was obtained from the individual(s) for the publication of any potentially identifiable images or data included in this article.

## Author contributions

NRA: Data curation, Formal analysis, Investigation, Methodology, Resources, Validation, Visualization, Writing – original draft, Writing – review & editing, Conceptualization, Supervision. AD: Conceptualization, Data curation, Formal analysis, Resources, Supervision, Validation, Visualization, Writing – review & editing. DR: Data curation, Formal analysis, Investigation, Methodology, Resources, Visualization, Writing – original draft. RS: Data curation, Investigation, Resources, Visualization, Writing – original draft. BC: Data curation, Writing – review & editing. DAC: Conceptualization, Data curation, Formal Analysis, Funding acquisition, Investigation, Methodology, Resources, Supervision, Validation, Visualization, Writing – review & editing.
